# Tree Height and the Asymptotic Mean of the Colijn–Plazzotta Rank of Unlabeled Binary Rooted Trees

**DOI:** 10.1007/s11538-025-01538-7

**Published:** 2025-11-03

**Authors:** Luc Devroye, Michael R. Doboli, Noah A. Rosenberg, Stephan Wagner

**Affiliations:** 1 School of Computer Science, McGill University, Montréal, Canada; 2 Department of Biology, Stanford University, Stanford, California USA; 3 Institute of Discrete Mathematics, TU Graz, Graz, Austria; 4 Department of Mathematics, Uppsala University, Uppsala, Sweden

**Keywords:** Colijn-Plazzotta rank, Mathematical phylogenetics, Tree height

## Abstract

The Colijn–Plazzotta ranking is a bijective encoding of the unlabeled binary rooted trees with positive integers. We show that the rank *f*(*t*) of a tree *t* is closely related to its height *h*, the maximal path length from a leaf to the root. We consider the rank $f(\tau _n)$ of a random *n*-leaf tree $\tau _n$ under each of three models: (i) uniformly random unlabeled unordered binary rooted trees, or unlabeled topologies; (ii) uniformly random leaf-labeled binary trees, or labeled topologies under the uniform model; and (iii) random binary search trees, or labeled topologies under the Yule–Harding model. Relying on the close relationship between tree rank and tree height, we obtain results concerning the asymptotic properties of $\log \log f(\tau _n)$. In particular, we find ${\mathbb {E}}\{\log _2 \log f(\tau _n)\} \sim 2 \sqrt{\pi n}$ for uniformly random unlabeled ordered binary rooted trees and uniformly random leaf-labeled binary trees, and for a constant $\alpha \approx 4.31107$, ${\mathbb {E}}\{\log _2 \log f(\tau _n)\} \sim \alpha \log n$ for leaf-labeled binary trees under the Yule–Harding model. We show that the mean of $f(\tau _n)$ itself under the three models is largely determined by the rank $c_{n-1}$ of the highest-ranked tree—the caterpillar—obtaining an asymptotic relationship with $\pi _n c_{n-1}$, where $\pi _n$ is a model-specific function of *n*. The results resolve open problems, providing a new class of results on an encoding useful in mathematical phylogenetics.

## Introduction

The Colijn–Plazzotta rank *f*(*t*) of a binary rooted tree *t* is defined recursively as follows (Colijn and Plazzotta 2018): if $\ell (t)$ and *r*(*t*) are the left and right subtree, respectively, arranged in such a way that $f\big (\ell (t)\big ) \ge f\big (r(t)\big )$, then

$$\begin{aligned} f(t) = \frac{f\big (\ell (t)\big ) \, \big (f(\ell (t))-1\big )}{2} + 1 + f\big (r(t)\big ). \end{aligned}$$

The rank 1 is assigned to a tree with a single leaf.

In the study of evolutionary trees, statistical summaries of trees are often used for characterizing the outcomes of evolutionary models and for statistical inference of the processes that have given rise to the trees (Fischer et al. 2023). Colijn–Plazzotta rank, or *CP rank*, has been used as a summary of tree shape in empirical scenarios in which trees of biological relationships are unconcerned with leaf labels, such as in examples with trees of sequences from the same pathogenic organism (Colijn and Plazzotta 2018).

Informally, for a fixed number of leaves, the CP rank is lowest for balanced trees and greatest for unbalanced trees. It has therefore been proposed as a measure of tree balance (Fischer et al. 2023; Rosenberg 2021). In a compilation of mathematical results for tree balance indices that capture many different features of rooted trees, Fischer et al. (2023) have listed a set of basic properties that are of interest for any balance index. Among these are the minimal and maximal values of the index across all trees with a fixed number of leaves, and the mean and variance of the index under the two most frequently used probabilistic models in mathematical phylogenetics. One is the uniform model, also sometimes known as the proportional-to-distinguishable-arrangements or PDA model, which assigns equal probability to all binary rooted labeled trees with a fixed number of leaves. The other is the Yule–Harding model, also sometimes known as the equal-rates Markov or ERM model or simply as the Yule model, in which, conditional on the number of leaves, the probability of a binary rooted labeled tree is proportional to the number of sequences of bifurcations that can give rise to the tree. The mathematical properties of balance indices assist in characterizing the way that balance indices relate to one another and how they perform in empirical settings.

The trees of minimal and maximal CP rank for a fixed number of leaves have been characterized (Rosenberg 2021), and indeed the asymptotic CP ranks of these trees in terms of the number of leaves have also been obtained (Doboli et al. 2024; Rosenberg 2021). The mean and variance under the uniform and Yule–Harding models have been listed as open problems (Fischer et al. 2023, p. 243).

We show here that the asymptotic mean and variance under the Yule–Harding model can be obtained by a connection between this model in the phylogenetics setting and the nearly equivalent formulation of random binary search trees in computer science. First, we show that the order of magnitude of the CP rank of a tree is determined by the height of the tree, the greatest distance from the root to a leaf. By connecting the CP rank to tree height and in turn to probabilistic results for the height, we obtain distributional properties of the CP rank under the Yule–Harding model. We also obtain related results on the closely related uniform model on labeled binary rooted trees and the uniform model on *un*labeled binary rooted trees.

## Tree Height and the Colijn–Plazzotta Rank

**Fig. 1:**
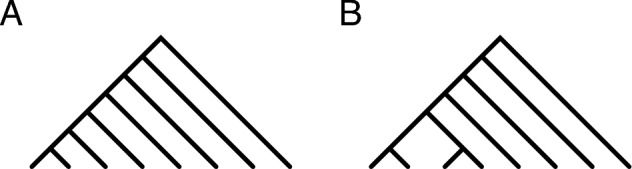
Caterpillar and pseudocaterpillar trees. (A) Caterpillar tree with $n=8$ leaves. The height of the tree is $n-1=7$. (B) Pseudocaterpillar tree with $n=8$ leaves. The height of the tree is $n-2=6$

We consider all trees to be binary and rooted. The *height* of a tree is the maximal path length in edges from the root to a leaf. Two special families of binary trees with *n* leaves play a key role in our analysis: the caterpillars, and the pseudocaterpillars (Figure 1). In a *caterpillar* with *n* leaves, $n \ge 1$, every non-leaf has at least one leaf child. This condition forces each caterpillar to consist of a chain of $n-1$ internal (i.e. non-leaf) nodes to which a layer of external nodes is added. The *pseudocaterpillars* (Rosenberg 2007) (or *4-pseudocaterpillars* in the terminology of Alimpiev and Rosenberg (2021)) can be constructed as follows for $n \ge 4$: start with a chain of $n-3$ internal nodes. Give the bottom node in the chain two children, and finally, complete the tree by adding a layer of *n* external nodes. Caterpillars have height $n-1$, and pseudocaterpillars have height $n-2$.

Among binary rooted trees with a fixed number of leaves, Rosenberg (2021, Corollary 10) found that the tree with the largest CP rank was the caterpillar. The CP rank of the caterpillar tree with *n* leaves can be computed recursively via a sequence termed $b_n$ by Rosenberg (2021, Theorem 9). It is convenient to shift the index of the sequence by 1 so that here, we will use $c_k$ to correspond to the CP rank of the caterpillar with height *k* and $k+1$ leaves. The sequence $c_k$ begins 1, 2, 3, 5, 12, 68, 2280 starting at $k=0$, matching OEIS A108225 (OEIS Foundation Inc. 2025) for $k \ge 1$.

### Lemma 1

Let the sequence $c_k$ be defined by $c_0 = 1$ and $c_{k+1} = c_{k}(c_{k}-1)/2 + 2$ for $k \ge 0$. For every tree *t* of height *h*, we have

$$\begin{aligned} c_h \le f(t) < c_{h+1}. \end{aligned}$$

### Proof

The proof proceeds by induction on *h*. For $h=0$, the tree consists of a single leaf, and we have $1 = c_0 = f(t) < c_1 = 2$. Thus, the statement holds in this case, and we can proceed with the induction step.

For a tree *t* of height *h*, suppose $h_{\ell } < h$ and $h_r < h$ are the heights of subtrees $\ell (t)$ and *r*(*t*), respectively. From the induction hypothesis for trees of height less than *h* and the left–right arrangement so that $f\big ( \ell (t) \big ) \ge f\big (r(t) \big )$, it follows that

$$\begin{aligned} c_{h_r} \le f(r(t)) \le f(\ell (t)) < c_{h_{\ell }+1}. \end{aligned}$$

The sequence $c_k$ is increasing (Rosenberg 2021, Lemma 8), so that $h_r < h_\ell + 1$, and hence, $h_r \le h_{\ell }$.

Because $h = \max (h_{\ell },h_r) + 1$, it follows that $h_{\ell } = h - 1$. Thus, we have, again by the induction hypothesis,

$$\begin{aligned} f(t)&= \frac{f\big (\ell (t)\big ) \, \big (f\big (\ell (t)\big )-1\big )}{2} + 1 + f\big (r(t)\big ) \\&\ge \frac{f\big (\ell (t)\big ) \, \big (f\big (\ell (t)\big )-1\big )}{2} + 1 + 1 \\&\ge \frac{c_{h-1}(c_{h-1}-1)}{2} + 2 \\&= c_h, \end{aligned}$$

which proves the lower bound. On the other hand,

$$\begin{aligned} f(t)&= \frac{f\big (\ell (t)\big ) \, \big (f(\ell (t))-1 \big )}{2} + 1 + f\big (r(t)\big ) \\&\le \frac{f\big (\ell (t)\big ) \, \big (f(\ell (t))-1\big )}{2} + 1 + f\big (\ell (t)\big ) \\&= \frac{f\big (\ell (t)\big ) \, \big (f\big (\ell (t)\big )+1\big )}{2} + 1 \\&\le \frac{(c_h-1)c_h}{2} + 1 \\&= c_{h+1} - 1, \end{aligned}$$

proving the upper bound. This completes the induction. $\square$

We conclude that the behavior of the height is to a great extent responsible for the behavior of the Colijn–Plazzotta rank of a tree. Indeed, because the CP rank is bijective with the positive integers (Rosenberg 2021, Proposition 2), the lemma implies that as the positive integers are traversed, for each $h\ge 0$, the ranking proceeds through trees with height *h*, then proceeds to those with height $h+1$, and so on. We immediately obtain the following corollaries (which are well known, see Harary et al. (1992)).

### Corollary 2

For $h\ge 0$, the number of unlabeled binary rooted trees with height at most *h* is $c_{h+1}-1$.

### Corollary 3

For $h\ge 0$, the number of unlabeled binary rooted trees with height exactly *h* is $c_{h+1}-c_h$.

The sequence $c_{h+1}-1$ begins at $h=0$ with values 1, 2, 4, 11, 67, 2279 (OEIS A006894). The sequence $c_{h+1}-c_h$ begins at $h=0$ with values 1, 1, 2, 7, 56, 2212 (OEIS A002658).

According to Rosenberg (2021, Corollary 14), $c_k \sim 2 \gamma ^{2^k}$ for a constant $\gamma \approx 1.11625$ as $k \rightarrow \infty$; note that $\gamma = \beta ^2$ for the constant $\beta$ in Rosenberg (2021), owing to the shift by 1 in $c_k$ relative to the indexing in Rosenberg (2021). We immediately obtain the following result.

### Corollary 4

Uniformly over all trees *t* with height *h*, we have,

$$\begin{aligned} 2^h + O(1) \le \log _{\gamma } f(t) \le 2^{h+1} + O(1), \end{aligned}$$

and thus, for $h > 0$,

$$\begin{aligned} \log _2 \log _{\gamma } f(t) = \log _2 \log f(t) + O(1) = h + O(1). \end{aligned}$$

In other words, the difference $|\log _2 \log _{\gamma } f(t) - h|$ is bounded by a universal constant.

We now analyze the behavior of the CP rank of random trees, which is mainly determined by the height. Indeed, we proceed by making use of extensive probabilistic results available on tree height under different sets of assumptions.

## Uniformly Random Unlabeled Binary Trees

Consider an unlabeled binary rooted tree on *n* leaves. Each node possesses either 0 offspring (leaves) or 2 offspring (internal nodes). Note that binary trees in which each node possesses either 0 or 2 (and not 1) offspring are sometimes termed *full* binary trees; here, all binary trees are “full” except where specified. A distinction exists between binary trees in which the left–right order of the children matters (*ordered* binary trees), and those in which the order is irrelevant (*unordered* binary trees, or *unlabeled topologies* in the terminology of mathematical phylogenetics, or *Otter trees* after Otter (1948)).

Let $\tau _n$ be a uniformly random ordered binary tree on $n \ge 1$ leaves, also called a random *Catalan tree* because the number of such trees is

$$ {k}_{n-1} = \frac{1}{n} \left( {\begin{array}{c}2n-2\\ n-1\end{array}}\right) , $$

where ${k}_n$ is the *n*-th Catalan number (Stanley 2015, Exercise 5). Catalan trees, viewed as ordered binary trees with *n* leaves, in which each node has 0 or 2 offspring, can be placed in bijection with trees with $n-1$ nodes in which the left–right order matters and each node has 0, 2, *or 1* offspring. For the bijection, we consider the latter type of tree, treating its $n-1$ nodes as internal nodes, and add descendant leaves so that each node that started with 0 or 1 offspring now has 2 offspring. Catalan trees are an example of a *simply generated family of trees*, and the random Catalan tree is also a special case of a *conditioned Galton–Watson tree*, with an offspring distribution whose support is $\{0,2\}$. See, for example, Sedgewick and Flajolet (1996, p. 224) and Drmota (2009, Section 1.2.7). We denote the CP rank of a random Catalan tree by $C_n$ (*C* for Catalan).

Let $\tau '_n$ be a uniformly random unordered binary tree, a uniformly random Otter tree. The number of such trees can be calculated recursively. The exact value ${u}_n$ (Wedderburn–Etherington number, OEIS A001190) for the number of such trees on *n* leaves follows

1 $$\begin{aligned} {u}_n = {\left\{ \begin{array}{ll} 1, & n=1, \\ \sum _{j=1}^{(n-1)/2} {u}_j {u}_{n-j}, & \text {odd } n \ge 3, \\ \bigg ( \sum _{j=1}^{n/2 - 1} {u}_j {u}_{n-j} \bigg ) + \frac{{u}_{n/2} ({u}_{n/2} + 1)}{2}, & \text {even } n \ge 2. \end{array}\right. } \end{aligned}$$

The asymptotic approximation follows (Harding 1971; Otter 1948)

2 $$\begin{aligned} {u}_n \sim \big (1+o(1)\big ) \frac{1}{{\kappa } n^{3/2} \rho ^n}, \end{aligned}$$

where ${\kappa } \approx 3.13699$ and $\rho \approx 0.40270$. The CP rank of a random Otter tree is denoted by $O_n$ (*O* for Otter).

To understand Theorem 5, we define a *theta random variable* as a random variable with distribution function (Devroye 1997)

3 $$\begin{aligned} F(x) = \frac{4 \pi ^{5/2}}{x^3} \sum _{j=1}^\infty j^2 e^{-\pi ^2 j^2/x^2} = \sum _{j=-\infty }^\infty (1-2j^2x^2) e^{-j^2 x^2}~, x > 0. \end{aligned}$$

CP rank is defined for unordered binary trees. To extend the CP rank to ordered binary trees, we compute the CP rank of the unordered binary tree associated with an ordered binary tree.

### Theorem 5

(i) Let $\tau _n$ be a uniformly random unlabeled binary tree with *n* leaves, with CP rank $C_n=f(\tau _n)$. Then
  $$ {\mathbb {E}}\{ \log _2 \log C_n \} \sim 2 \sqrt{\pi n}, $$
  and
  $$ \frac{\log _2 \log C_n}{2\sqrt{n}} $$
  converges in distribution to a theta random variable as defined by (3).
(ii) Let $\tau '_n$ be a uniformly random unlabeled unordered binary tree with *n* leaves, with CP rank $O_n=f(\tau '_n)$. Then, with $\kappa$ as in (2),
  $$ {\mathbb {E}}\{ \log _2 \log O_n \} \sim \kappa \sqrt{n}, $$
  and
  $$ \frac{\log _2 \log O_n}{\kappa \sqrt{n/\pi }} $$
  converges in distribution to a theta random variable as defined by (3).

### Proof

(i) The statement on $\tau _n$ is a consequence of a result of Flajolet and Odlyzko (1982, Theorem B) about the height $H_n$ of $\tau _n$: ${\mathbb {E}}\{ H_n \} /\sqrt{n} \rightarrow 2 \sqrt{\pi }$ as $n \rightarrow \infty$, and ${H_n }/{(2\sqrt{n})}$ tends in distribution to a theta random variable. By Corollary 4, the difference $|\log _2 \log C_n - H_n|$ is (deterministically, thus almost surely) bounded by a universal constant, so that
  $$\begin{aligned} \frac{\log _2 \log C_n - H_n}{2\sqrt{n}} \end{aligned}$$
  is $O(n^{-1/2})$; for any sequence of random trees of increasing size, this quantity goes to 0 (almost sure convergence, and hence, convergence in probability). The statement on the expected value now follows from the linearity of expectation and the statement on convergence in distribution follows from Slutsky’s theorem applied to the convergence in distribution of ${H_n }/{(2\sqrt{n})}$ and the convergence in probability to 0 of $(\log _2 \log C_n - H_n)/(2\sqrt{n})$.
(ii) The statement on $\tau '_n$ follows in the same fashion from the results of Broutin and Flajolet (2008, Theorem 1 and Theorem 5; 2012, Theorem 1 and Theorem 3) on the height of unlabeled unordered binary trees. These state that the height $H'_n$ of a random unlabeled unordered binary tree with *n* leaves satisfies ${\mathbb {E}}\{ H'_n \} /\sqrt{n} \rightarrow \kappa$, and that ${H'_n }/{(\kappa \sqrt{n/\pi })}$ tends in distribution to a theta random variable. We remark here that our notation differs slightly from Broutin and Flajolet (2012): our constant $\kappa \approx 3.13699$ corresponds to the constant denoted $2 \sqrt{\pi }/\lambda$ in Broutin and Flajolet (2012), and our distribution function *F*(*x*) in (3) is $1-\Theta (2x)$ in the notation of Broutin and Flajolet. $\square$

## Uniformly Random Leaf-Labeled Binary Trees

A leaf-labeled binary tree with *n* leaves is a binary tree in which the leaves are bijectively labeled from 1 to *n*, and in which each internal node has two children. The children are unordered. Such trees are also called *labeled topologies* or *cladograms*.

We consider a uniformly random cladogram $\tau _n$. The number of such trees is

4 $$\begin{aligned} (2n-3)\cdot (2n-5)\cdots 3\cdot 1 = \frac{1}{2^{n-1}} \, \frac{(2n-2)!}{(n-1)!}, \end{aligned}$$

all of which are equally likely under this model of randomness (OEIS A001147). The CP rank of a random cladogram is denoted by $L_n$ (*L* for labeled).

A model of uniformly random cladograms is a special case of more general models on the cladograms, such as Ford’s alpha-splitting model (Ford 2005, 2006) and Aldous’s beta-splitting model (Aldous 1996, 2001). In particular, Aldous (1996, Proposition 4, $\beta =-\frac{3}{2}$ case) showed that the expected height of a random cladogram satisfies

$$ {\mathbb {E}}\left\{ H_n \right\} \sim 2 \sqrt{\pi n}. $$

It is worth pointing out that this result (including the constant $2\sqrt{\pi }$) is the same as for uniformly random unlabeled ordered binary trees (compare to Theorem 5i). This is no coincidence: for every unlabeled ordered binary tree on *n* leaves, there are *n*! possibilities to label the leaves and turn it into a leaf-labeled ordered binary tree. Likewise, precisely $2^{n-1}$ possibilities turn a labeled unordered binary tree on *n* leaves into a labeled ordered binary tree (by switching the order of the children at the internal nodes). For this reason, the distribution of the height and any other parameters that do not depend on labels or order is the same for three uniform models: unlabeled ordered, labeled unordered, and labeled ordered binary trees (Disanto et al. 2022, Section 3.1). In particular, the following result is equivalent to part (i) of Theorem 5.

### Theorem 6

Let $\tau _n$ be a uniformly random leaf-labeled binary tree with *n* leaves, with CP rank $L_n=f(\tau _n)$. Then

$$ {\mathbb {E}}\{ \log _2 \log L_n \} \sim 2 \sqrt{\pi n}, $$

and

$$ \frac{\log _2 \log L_n}{2\sqrt{n}} $$

converges to a theta distribution.

Aldous’s beta-splitting model for random binary trees has a shape parameter $\beta \in [-2, \infty ]$, encompassing a limiting unbalanced model ($\beta =-2$), a limiting balanced model ($\beta =\infty$), the Yule model ($\beta =0$), and the uniform model in Theorem 6 ($\beta = -\frac{3}{2}$). Generally, Aldous (1996, Proposition 4) proved the following results on the height $H_n$:

- For $\beta > -1$, the ratio $H_n / \log n$ tends in probability and in expectation to a constant $g(\beta )$. There is no explicit expression for this constant, but numerical values can be determined from an implicit equation given by Aldous (1996, Proposition 4). To mention some examples, $g(\infty ) = 1/\log 2 \approx 1.44270$, and we obtain $g(1) \approx 3.19258$, $g(0) \approx 4.31107$, and $g(-\frac{1}{2}) \approx 6.38090$ from the implicit equation (note that Aldous (1996) only gives two digits each). The case $\beta =0$ corresponds to the Yule model (see Section 5 below for more information). For $\beta = \infty$, all internal nodes split their subtrees (almost) precisely in half: the difference of the subtree sizes is at most 1.
- For $\beta = -1$, ${\mathbb {E}}\{ H_n \} \ge \big (6/\pi ^2 + o(1)\big )(\log n)^2$. Aldous’s proposition did not report a result for ${\mathbb {E}}\{ H_n \}$ with $\beta =-1$, but this inequality follows quickly from Aldous’s results reported in the proposition for related quantities. Recently, Aldous and Pittel (2025, Theorem 1.5) showed that $H_n \le (\gamma + \epsilon ) (\log n)^2$ with probability approaching 1 with increasing *n*, where $\epsilon > 0$ and $\gamma \approx 42.9$.
- For $\beta \in (-2,-1)$, $n^{1+\beta } {\mathbb {E}}\{H_n \} \rightarrow g(\beta )$, and $n^{1+\beta } H_n$ has a non-degenerate limit distribution.

These results on tree height for cladograms under the beta-splitting model directly impact the Colijn–Plazzotta rank. For example, for $\beta \in (-2,-1)$, we have for Aldous’s beta-splitting tree $\tau _n$ with *n* leaves

$$ {\mathbb {E}}\{ \log _2 \log f(\tau _n) \}\sim \frac{g(\beta )}{n^{1+\beta }} . $$

## Yule–Harding Trees, Random Binary Search Trees

Among the probability distributions that could be placed on the leaf-labeled binary trees with *n* leaves, perhaps the most frequently considered, along with the uniform distribution of Section 4, is the $\beta =0$ case of the beta-splitting model. This model corresponds to the random binary search trees, which are identical to Yule or Yule–Harding trees in phylogenetics (Fuchs 2025), except for the convention that random binary search trees are typically indexed by the number of internal nodes and Yule–Harding trees are indexed by the number of leaves. We index trees by the number of leaves, considering random binary search trees in which all internal nodes have two children so that the total number of internal nodes is $n-1$ when the number of leaves is *n*.

To be precise, we start with a standard random binary search tree on $n-1$ (internal) nodes and attach a layer of *n* external nodes, i.e., we give a second child to all (internal) nodes having one child, and give two children to all leaves. The random CP rank of a tree under this model is denoted by $S_n$ (*S* for search tree).

For these trees, the height $H_n$ satisfies (Devroye 1986, Theorem 5.1)

$$ \frac{H_n}{\log n} \xrightarrow {p} \alpha , $$

where $\alpha \approx 4.31107$ is the unique solution in $(2, \infty )$ of the equation

$$ \alpha \log (2e/\alpha ) = 1. $$

Setting

$$ \beta = \frac{3 \alpha }{2 \alpha -2 } \approx 1.95303, $$

Reed (2003) and Drmota (2003) showed that $H_n - \alpha \log n + \beta \log \log n$ is tight, i.e.,

5 $$\begin{aligned} \limsup _{x \uparrow \infty } \, \big [\sup _n {\mathbb {P}} \left\{ | H_n - \alpha \log n + \beta \log \log n | \ge x \right\} \big ] = 0. \end{aligned}$$

One way to see this result is as follows: Reed (2003, Theorem 1) states that

$$\begin{aligned} {\mathbb {E}}\{ H_n - \alpha \log n + \beta \log \log n \} = O(1) \end{aligned}$$

and

$$\begin{aligned} {\mathbb {V}}\{ H_n - \alpha \log n + \beta \log \log n \} = {\mathbb {V}}\{H_n\} = O(1), \end{aligned}$$

from which tightness follows by a standard application of the Chebyshev inequality. Alternatively, one can use Lemmas 8 and 10 of Reed (2003), which provide explicit tail bounds.

### Theorem 7

Let $\tau _n$ be a random leaf-labeled binary tree with *n* leaves following the Yule–Harding distribution, with CP rank $S_n=f(\tau _n)$. Then

$$\begin{aligned} {\mathbb {E}}\{\log _2 \log S_n \} \sim \alpha \log n, \end{aligned}$$

and

$$ \frac{\log _2 \log S_n}{\log n} \overset{p}{\rightarrow }\ \alpha . $$

### Proof

The proof is similar to Theorem 5. By Corollary 4, the difference between $\log _2 \log S_n$ and the height $H_n$ is bounded, so

$$ \frac{\log _2 \log S_n - H_n}{\log n} $$

goes to 0 (almost surely, thus also in probability). The second part of the result follows immediately via Slutsky’s theorem from the fact that $H_n / \log n \overset{p}{\rightarrow }\ \alpha$; the first part follows from the fact that ${\mathbb {E}}\{ H_n / \log n \} \rightarrow \alpha$ as $n \rightarrow \infty$ (Devroye 1986). $\square$

### Theorem 8

Let $\tau _n$ be a random leaf-labeled binary tree with *n* leaves following the Yule–Harding distribution, with CP rank $S_n=f(\tau _n)$. Then

$$ \frac{(\log n)^{\beta \log 2} \log S_n}{n^{\alpha \log 2}} $$

is a tight sequence of random variables.

### Proof

By Corollary 4, there exists an absolute positive constant *K* such that $K\cdot 2^{H_n} \ge \log S_n$. Thus,

$$\begin{aligned} \frac{(\log n)^{\beta \log 2} \log S_n}{n^{\alpha \log 2}} \ge x \end{aligned}$$

implies

$$\begin{aligned} 2^{H_n} \ge \frac{x n^{\alpha \log 2}}{K (\log n)^{\beta \log 2}}, \end{aligned}$$

or

$$\begin{aligned} H_n - \alpha \log n + \beta \log \log n \ge \frac{\log (x/K)}{\log 2}. \end{aligned}$$

This means that

$$\begin{aligned} {\mathbb {P}} \Big \{ \Big | \frac{(\log n)^{\beta \log 2} \log S_n}{n^{\alpha \log 2}} \Big | \ge x \Big \}&= {\mathbb {P}} \Big \{ \frac{(\log n)^{\beta \log 2} \log S_n}{n^{\alpha \log 2}} \ge x \Big \} \\&\le {\mathbb {P}} \Big \{ H_n - \alpha \log n + \beta \log \log n \ge \frac{\log (x/K)}{\log 2} \Big \} \\&\le {\mathbb {P}} \Big \{ |H_n - \alpha \log n + \beta \log \log n| \ge \frac{\log (x/K)}{\log 2} \Big \}. \end{aligned}$$

By (5), this expression goes to 0 if we take $\sup _n$ and then $\limsup _{x \uparrow \infty }$, showing that the sequence is indeed tight.

## Mean and Variance of the Colijn–Plazzotta Rank

Sections 3–5 focus on properties of the distribution of $\log \log f(\tau _n)$ under various models of randomness; in this section, we focus on the distribution of the random CP rank $f(\tau _n)$ itself. In particular, we study the first-order asymptotics of the mean and variance of the Colijn–Plazzotta rank under the models of randomness from Sections 3–5, investigating $C_n$, $O_n$, $L_n$, and $S_n$. As pointed out in Section 4, the models of uniformly random unlabeled ordered binary trees (Catalan trees) and uniformly random labeled unordered binary trees are equivalent for our purposes, so that the distributions of $C_n$ and $L_n$ are the same.

We give a general theorem on the mean and variance of the Colijn–Plazzotta rank applicable to all random tree models specifying a certain condition. We then obtain first-order asymptotics for the means and variances of $C_n$, $O_n$, $L_n$ and $S_n$ as simple corollaries. The desired means and variances are determined mainly by the extreme cases for Colijn–Plazzotta ranks.

### Lemma 9

(i) Among all unlabeled binary rooted trees with *n* leaves, $n \ge 1$, the Colijn–Plazzotta rank is maximized by the caterpillar. (ii) Among all unlabeled binary rooted trees with *n* leaves and height $n-2$ or less, $n \ge 4$, the Colijn–Plazzotta rank is maximized by the pseudocaterpillar.

### Proof

(i) This result was proven in Corollary 20 of Rosenberg (2021).
(ii) This result follows by induction and Lemma 1. For $n=4$, the pseudocaterpillar is the only tree with height at most $n-2=2$. Suppose for induction that for all *k*, $4 \le k \le n-1$, the pseudocaterpillar has the maximal Colijn–Plazzotta rank among trees with *k* leaves and height $k-2$. Among trees *t* with *n* leaves and height at most $n-2$, by definition of the Colijn–Plazzotta rank, the rank *f*(*t*) is maximized by choosing its left subtree $\ell (t)$ to have $f\big (\ell (t)\big )$ as large as possible. The left subtree $\ell (t)$ has at most $n-1$ leaves and height at most $n-3$, so that the inductive hypothesis applies: $\ell (t)$ is the pseudocaterpillar with $n-1$ leaves, the right subtree *r*(*t*) is a single leaf, and *t* is the pseudocaterpillar with *n* leaves. $\square$

For the following theorem, we recall Rosenberg’s (2021) sequence for the maximal Colijn–Plazzotta rank $c_h$ among trees with height $h \ge 0$ and $h+1$ leaves: $c_0 = 1$, and

6 $$\begin{aligned} c_{h+1} = \left( {\begin{array}{c}c_h\\ 2\end{array}}\right) + 2, \, h \ge 0. \end{aligned}$$

Equivalently, $c_h$ is the Colijn–Plazzotta rank of a caterpillar of height *h*. Recall that $c_2 = 3$, $c_3 = 5$, $c_4 = 12$, and $c_5 = 68$.

We also let $d_h$ be the corresponding rank of a pseudocaterpillar of height *h*. Then $d_2=4$, and

7 $$\begin{aligned} d_{h+1} = \left( {\begin{array}{c}d_h\\ 2\end{array}}\right) + 2, \, h \ge 2. \end{aligned}$$

The sequences $c_h$ and $d_h$ obey identical recursions, only with different starting points. Sequence $d_h$ begins with $d_2=4$, $d_3=8$, $d_4=30$, and $d_5=437$.

### Theorem 10

For a given probability model for random binary rooted trees $T_n$ with *n* leaves, let

$$ \pi _n = {\mathbb {P}} \left\{ T_n ~\text {is a caterpillar} \right\} , $$

and let $P_n$ be the Colijn–Plazzotta rank of $T_n$. If $\pi _n = o(1)$ and

8 $$\begin{aligned} \log (1/\pi _n) = o(2^n), \end{aligned}$$

then

$$\begin{aligned} {\mathbb {E}}\left\{ P_n \right\}&\sim \pi _n c_{n-1}, \\ {\mathbb {V}}\left\{ P_n \right\} \sim {\mathbb {E}}\left\{ P_n^2 \right\}&\sim \pi _n c_{n-1}^2. \end{aligned}$$

The idea of the result is that under the conditions specified, the CP rank of the *n*-leaf pseudocaterpillar—the tree of second-largest CP rank among those with *n* leaves—grows sufficiently slowly that the CP ranks of this tree and all other non-caterpillar trees are negligible in relation to that of the *n*-leaf caterpillar. The mean and variance of the CP rank of a random tree then depend only on the probability that a tree is a caterpillar and the CP rank of the caterpillar.

### Proof

We distinguish two events. If $T_n$ is a caterpillar of height $n-1$, then $P_n = c_{n-1}$. Otherwise, if $T_n$ is some other tree, then its CP rank $P_n$ has upper bound $d_{n-2}$, the CP rank of a pseudocaterpillar of height $n-2$. These values yield the trivial bounds

9 $$\begin{aligned}&\pi _n c_{n-1} \le {\mathbb {E}}\left\{ P_n \right\} \le \pi _n c_{n-1} + d_{n-2}, \end{aligned}$$

10 $$\begin{aligned}&\pi _n c_{n-1}^2 \le {\mathbb {E}}\left\{ P_n^2 \right\} \le \pi _n c_{n-1}^2 + d_{n-2}^2. \end{aligned}$$

By taking the ratio of (9) with $\pi _n c_{n-1}$, to verify ${\mathbb {E}}\left\{ P_n \right\} \sim \pi _n c_{n-1}$, it suffices to show

11 $$\begin{aligned} \lim _{n \rightarrow \infty } \frac{ d_{n-2}}{\pi _n c_{n-1}} = 0. \end{aligned}$$

Similarly, because $d_{n-2} < c_{n-1}$ so that $(d_{n-2} / c_{n-1})^2 < d_{n-2} / c_{n-1}$, by taking the ratio of (10) and $\pi _n c_{n-1}^2$, verifying condition (11) suffices for verifying ${\mathbb {V}}\left\{ P_n \right\} \sim {\mathbb {E}}\left\{ P_n^2 \right\} \sim \pi _n c_{n-1}^2$; we see first that ${\mathbb {E}}\left\{ P_n^2 \right\} \sim \pi _n c_{n-1}^2$, and then ${\mathbb {V}}\left\{ P_n \right\} ={\mathbb {E}}\left\{ P_n^2 \right\} - {\mathbb {E}}\left\{ P_n \right\} ^2 \sim {\mathbb {E}}\left\{ P_n^2 \right\}$ follows by recalling that $\pi _n = o(1)$.

We will show that (8) implies (11). We first prove by induction that $d_{h-1} < 0.9^{2^{h-3}} c_h$ for all $h \ge 3$. This statement is readily verified for $h = 3$ and $h=4$. Now assume that the inequality holds for some positive integer $h \ge 4$, and write $Q_h = 0.9^{-(2^{h-3})} > 1$, so that $c_h > Q_h d_{h-1}$. It follows from the recursions (6) and (7) that

$$\begin{aligned} \frac{d_h}{c_{h+1}}&= \frac{d_{h-1}^2 - d_{h-1} + 4}{c_h^2 - c_h + 4} < \frac{d_{h-1}^2 - d_{h-1} + 4}{Q_h^2 d_{h-1}^2 - Q_h d_{h-1} + 4} \\&= \frac{1}{Q_h^2} - \frac{(Q_h-1)(Q_h d_{h-1}-4Q_h-4)}{Q_h^2(Q_h^2 d_{h-1}^2 - Q_h d_{h-1} + 4)}. \end{aligned}$$

The final fraction is positive since $Q_h > 1$ and $d_{h-1} \ge d_3 \ge 8$. Thus,

$$\begin{aligned} \frac{d_h}{c_{h+1}} < \frac{1}{Q_h^2} = 0.9^{2^{h-2}}, \end{aligned}$$

completing the induction.

It follows (for $n \ge 4$) that

$$\log \frac{d_{n-2}}{\pi _n c_{n-1}} \le \log \Big ( \frac{1}{\pi _n} 0.9^{2^{n-4}}\Big ) = 2^{n-4} \log 0.9 - \log \pi _n = 2^{n-4} \log 0.9 + o(2^n)$$

by the assumption (8). Because this last expression goes to $-\infty$ as *n* increases without bound, we have verified (11). This completes the proof. $\square$

The theorem finds that the asymptotic mean is simply the product of the CP rank of the caterpillar and the probability that a tree is a caterpillar. In all four types of random trees that we consider, we verify that $\pi _n$ satisfies (8), so that the theorem applies. This verification amounts to demonstrating that caterpillars are sufficiently probable as *n* grows large; if $\pi _n$ were to decrease too quickly, then the condition would not be satisfied.

**Table 1:** CP rank $f(t_n)$ and probability under three models for all unlabeled unordered binary trees $t_n$ with *n* leaves, $1 \le n \le 7$. For unlabeled uniform unordered trees, the probability is the reciprocal of the number of such trees, the Wedderburn–Etherington number defined by (1). For leaf-labeled uniform trees, it is the ratio of $n! / 2^{s(t_n)}$ (the number of ways of labeling shape $t_n$, where the number of symmetric nodes $s(t_n)$ is the number of internal nodes whose two descendant subtrees have the same unlabeled shape) and $(2n-3)!!$, the number of leaf-labeled trees with *n* leaves (4). For leaf-labeled Yule–Harding trees, it is the ratio of $[n! / 2^{s(t_n)}][(n-1)!/\prod _{r=2}^n (r-1)^{d_r(t_n)}]$ and $n!(n-1)!/2^{n-1}$, where $d_r(t_n)$ is the number of internal nodes of $t_n$ with *r* descendant leaves, $(n-1)!/\prod _{r=2}^n (r-1)^{d_r(t_n)}$ gives the number of *labeled histories* of a leaf-labeled tree (the number of sequences in which the tree can be produced by a sequence of bifurcations), and $n!(n-1)!/2^{n-1}$ is the total number of labeled histories for *n* labeled leaves

				Model		
*n*	$t_n$	$f(t_n)$	Height	Unlabeled uniform unordered	Leaf-labeled uniform	Leaf-labeled Yule–Harding
1		1	0	1	1	1
2		2	1	1	1	1
3		3	2	1	1	1
4		5	3	1/2	4/5	2/3
4		4	2	1/2	1/5	1/3
5		12	4	1/3	4/7	1/3
5		8	3	1/3	1/7	1/6
5		6	3	1/3	2/7	1/2
6		68	5	1/6	8/21	2/15
6		30	4	1/6	2/21	1/15
6		17	4	1/6	4/21	1/5
6		13	4	1/6	4/21	4/15
6		9	3	1/6	1/21	2/15
6		7	3	1/6	2/21	1/5
7		2280	6	1/11	8/33	2/45
7		437	5	1/11	2/33	1/45
7		138	5	1/11	4/33	1/15
7		80	5	1/11	4/33	4/45
7		38	4	1/11	1/33	2/45
7		23	4	1/11	2/33	1/15
7		69	5	1/11	4/33	1/9
7		31	4	1/11	1/33	1/18
7		18	4	1/11	2/33	1/6
7		14	4	1/11	4/33	2/9
7		10	3	1/11	1/33	1/9

**Table 2:** CP rank $f(t_n)$ and probability under three models for all unlabeled unordered binary trees $t_n$ with *n* leaves, $n=8$. The table design follows Table 1

				Model		
*n*	$t_n$	$f(t_n)$	Height	Unlabeled uniform unordered	Leaf-labeled uniform	Leaf-labeled Yule–Harding
8		2598062	7	1/23	64/429	4/315
8		95268	6	1/23	16/429	2/315
8		9455	6	1/23	32/429	2/105
8		3162	6	1/23	32/429	8/315
8		705	5	1/23	8/429	4/315
8		255	5	1/23	16/429	2/105
8		2348	6	1/23	32/429	2/63
8		467	5	1/23	8/429	1/63
8		155	5	1/23	16/429	1/21
8		93	5	1/23	32/429	4/63
8		47	4	1/23	8/429	2/63
8		2281	6	1/23	32/429	4/105
8		438	5	1/23	8/429	2/105
8		139	5	1/23	16/429	2/35
8		81	5	1/23	16/429	8/105
8		39	4	1/23	4/429	4/105
8		24	4	1/23	8/429	2/35
8		70	5	1/23	32/429	2/21
8		32	4	1/23	8/429	1/21
8		19	4	1/23	16/429	1/7
8		16	4	1/23	16/429	4/63
8		15	4	1/23	8/429	4/63
8		11	3	1/23	1/429	1/63

**Table 3:** Summary of the main asymptotic results under three models. $\tau _n$ refers to a random tree with *n* leaves under the model, and $f(\tau _n)$ is the associated random CP rank. Properties of random trees are the same for uniformly random leaf-labeled unordered trees and for uniformly random unlabeled ordered trees

	Model		
Property	Unlabeled	Leaf-labeled	Leaf-labeled
	uniform unordered	uniform	Yule–Harding
${\mathbb {E}}\{\log \log f(\tau _n)\}$	Theorem 5	Theorem 6	Theorem 7
$\log f(\tau _n)$	-	-	Theorem 8
${\mathbb {E}}\{f(\tau _n)\}$	Theorem 10	Theorem 10	Theorem 10
${\mathbb {V}}\{f(\tau _n)\}$	Theorem 10	Theorem 10	Theorem 10

**Fig. 2:**
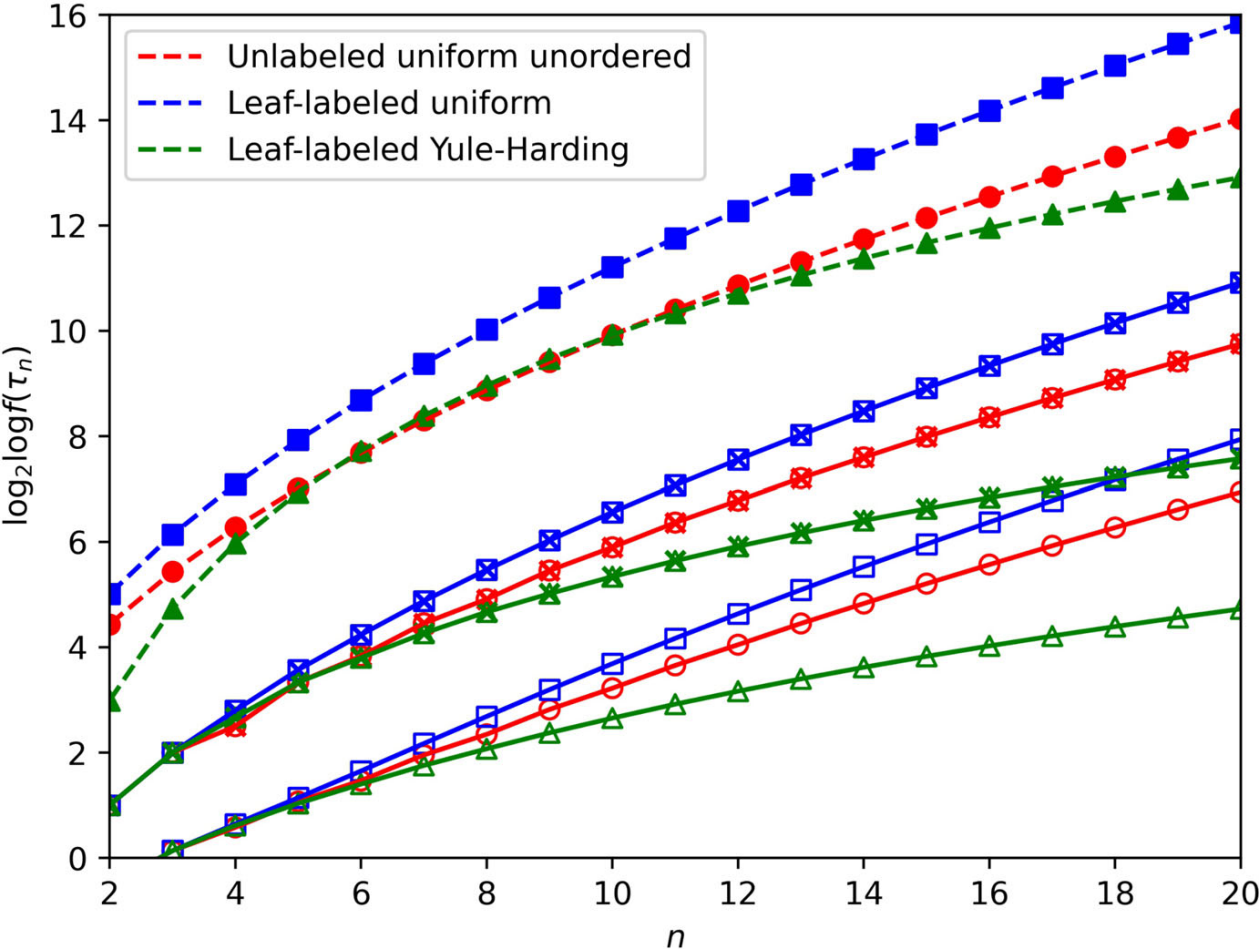
Expected value of the double logarithm of CP rank, ${\mathbb {E}}\{ \log _2 \log f(\tau _n) \}$, under three models, for $n=2$ to 20: uniformly random unlabeled unordered binary trees, uniformly random leaf-labeled binary trees, and Yule–Harding leaf-labeled binary trees. Exact values of ${\mathbb {E}}\{ \log _2 \log f(\tau _n) \}$ (open symbols) appear alongside exact values of the expected tree height ${\mathbb {E}}\{ H_n \}$ (open symbols superimposed with crosses) under the three models and the asymptotic expressions (closed symbols, dashed lines): $\kappa \sqrt{n}$ for unlabeled uniform unordered (Theorem 5ii), $2 \sqrt{\pi n}$ for leaf-labeled uniform (Theorem 6), and $\alpha \log n$ for leaf-labeled Yule–Harding (Theorem 7). $\kappa \approx 3.13699$, $\alpha \approx 4.31107$ (color figure online)

**Fig. 3:**
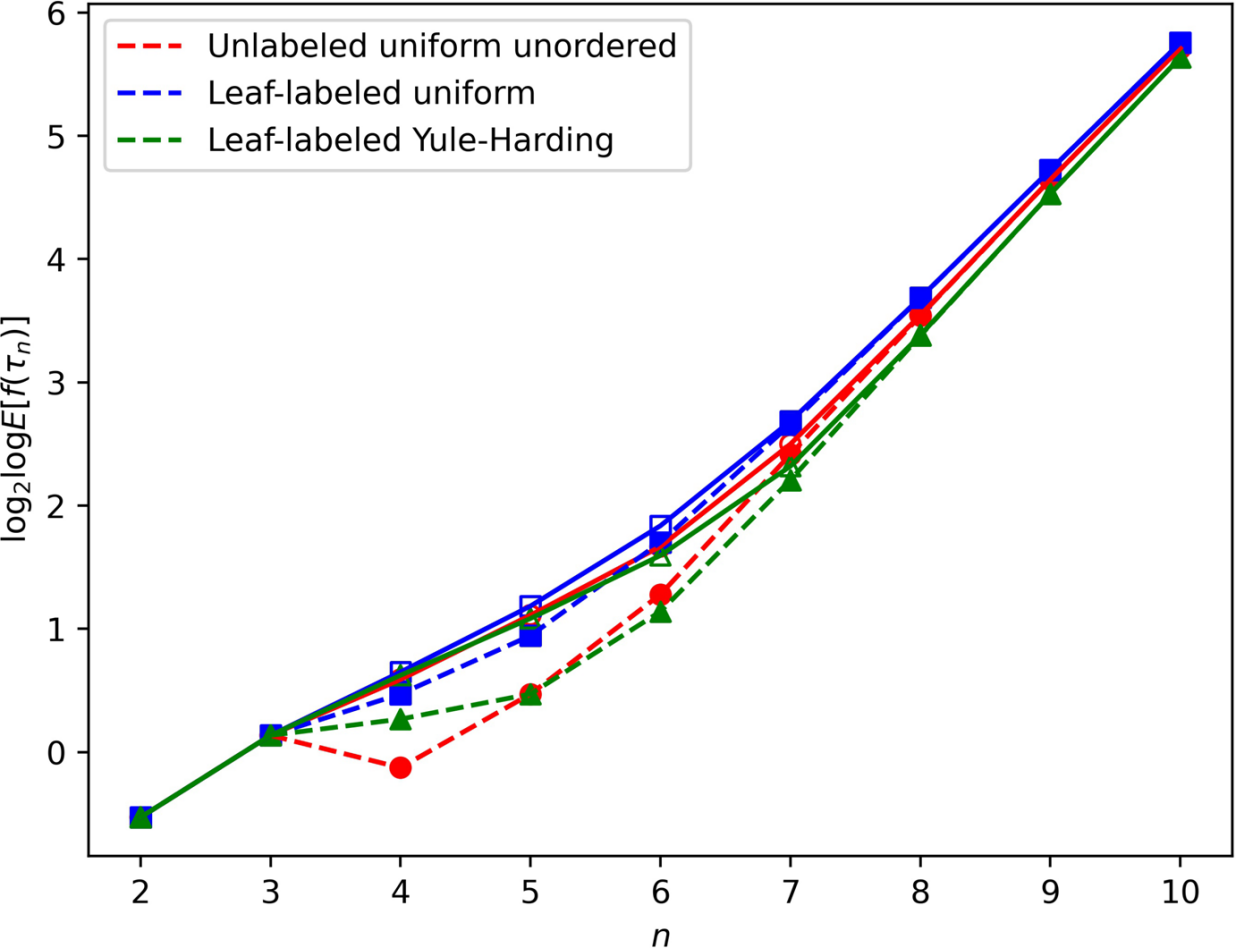
Expected value of the CP rank, ${\mathbb {E}}\{ f(\tau _n) \}$, under three models, for $n=2$ to 10: uniformly random unlabeled unordered binary trees, uniformly random leaf-labeled binary trees, and Yule–Harding leaf-labeled binary trees. Exact values of $\log _2 \log {\mathbb {E}}\{ f(\tau _n) \}$ (open symbols) appear alongside asymptotic expressions $\log _2 \log (\pi _n c_{n-1})$ from Theorem 10 (closed symbols, dashed lines), where $\pi _n$ follows (12) for leaf-labeled uniform and (14) for leaf-labeled Yule–Harding and $c_{n-1}$ is the CP rank of the caterpillar with $n-1$ internal nodes and *n* leaves (6). For unlabeled uniform unordered, $\pi _n$ is computed as the exact $1/u_n$, where $u_n$ is the Wedderburn–Etherington number defined by (1) (color figure online)

**Fig. 4:**
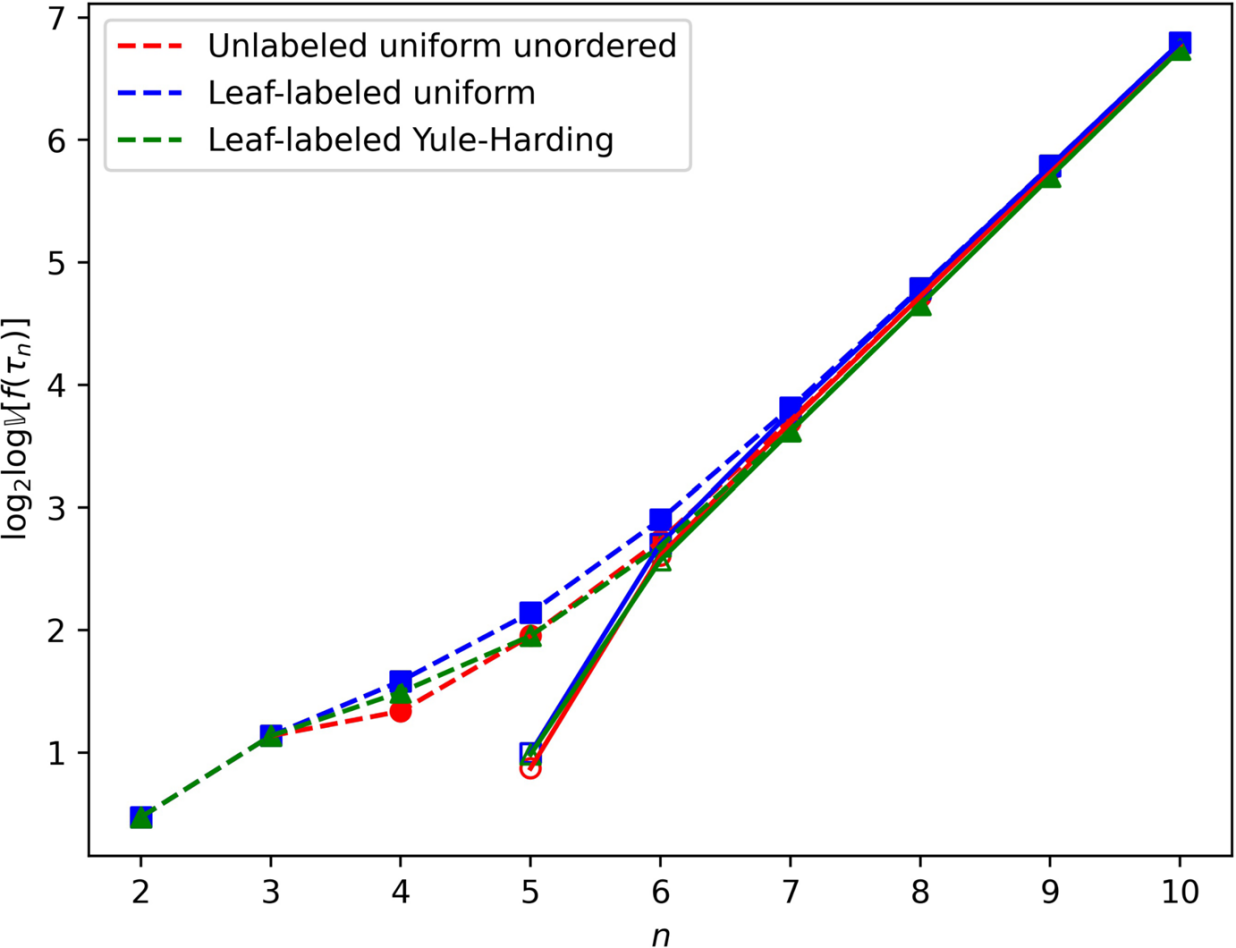
Variance of the CP rank, ${\mathbb {V}}\{ f(\tau _n) \}$, under three models, for $n=2$ to 10: uniformly random unlabeled unordered binary trees, uniformly random leaf-labeled binary trees, and Yule–Harding leaf-labeled binary trees. Exact values of $\log _2 \log {\mathbb {V}}\{ f(\tau _n) \}$ (open symbols) appear alongside asymptotic expressions $\log _2 \log (\pi _n c_{n-1}^2)$ from Theorem 10 (closed symbols, dashed lines), where $\pi _n$ follows (12) for leaf-labeled uniform and (14) for leaf-labeled Yule–Harding and $c_{n-1}$ is the CP rank of the caterpillar with $n-1$ internal nodes and *n* leaves (6). For unlabeled uniform unordered, $\pi _n$ is computed as the exact $1/u_n$, where $u_n$ is the Wedderburn–Etherington number defined by (1) (color figure online)

The number of caterpillar cladograms is *n*!/2, so that for a random cladogram (and equivalently, for a random Catalan tree), (4) gives

12 $$\begin{aligned} \pi _n = \frac{n!}{2} \frac{1}{(2n-3)!!} = \frac{2^{n-2}}{\frac{1}{n} \left( {\begin{array}{c}2n-2\\ n-1\end{array}}\right) } \sim \frac{2^{n-2}}{\pi ^{-1/2}n^{-3/2}4^{n-1}} \sim \frac{n^{3/2} \sqrt{\pi }}{2^n}. \end{aligned}$$

For a random Otter tree on *n* leaves, we have no simple explicit expression for $\pi _n$. However, we have the asymptotic probability from (2) that a random Otter tree is the unique caterpillar:

13 $$\begin{aligned} \pi _n {=\frac{1}{u_n} \sim \kappa n^{3/2} \rho ^{n}}. \end{aligned}$$

Finally, for a random binary search tree (Slowinski 1990, p. 92),

14 $$\begin{aligned} \pi _n = \frac{n!}{2} \frac{1}{\frac{n! \, (n-1)!}{2^{n-1}}} = \frac{2^{n-2}}{(n-1)!} \sim \bigg ( \frac{2e}{n} \bigg )^n \frac{\sqrt{n}}{4\sqrt{2\pi }}. \end{aligned}$$

Verifying in (12), (13), and (14) that condition (8) is satisfied, we have shown the following theorem.

### Theorem 11

With $\pi _n$ as in (12), (13), and (14), and with $P_n$ corresponding to either $C_n$ (the random Catalan tree), $O_n$ (the random Otter tree), $L_n$ (the random cladogram), or $S_n$ (the random binary search tree), we have

$$\begin{aligned} {\mathbb {E}}\left\{ P_n \right\}&\sim \pi _n c_{n-1}, \\ {\mathbb {V}}\left\{ P_n \right\} \sim {\mathbb {E}}\left\{ P_n^2 \right\}&\sim \pi _n c_{n-1}^2. \end{aligned}$$

## Numerical Computations

We informally examine the extent to which the asymptotic approximations for ${\mathbb {E}}\{\log _2 \log f(\tau _n)\}$, ${\mathbb {E}}\{ f(\tau _n) \}$, and ${\mathbb {V}}\{ f(\tau _n) \}$ agree with the exact values for small *n*. First, Tables 1 and 2 show the CP rank and the probabilities of all unlabeled unordered binary trees for $n=1$ to 8 under each of three models: uniformly random unlabeled unordered trees, uniformly random leaf-labeled trees, and Yule–Harding leaf-labeled trees. The much larger CP rank for the caterpillar compared to the pseudocaterpillar (and all other trees) is already visible for $n=8$.

Figure 2 plots the values of ${\mathbb {E}}\{\log _2 \log f(\tau _n)\}$, the mean height $H_n$, and the asymptotic approximation for ${\mathbb {E}}\{\log _2 \log f(\tau _n)\}$ under the three models. For each of the three models, we can observe similar shapes in plots for its three quantities. The values are greatest for the uniformly random leaf-labeled trees, with asymptotic approximation $2 \sqrt{\pi n} \approx 3.54491 \sqrt{n}$, followed by the uniformly random unlabeled unordered trees, with asymptotic approximation $3.13699 \sqrt{n}$, and finally, the Yule–Harding leaf-labeled trees, with asymptotic approximation $4.31107 \log n$.

Figures 3 and 4 plot the exact mean and variance of $f(\tau _n)$ under the three models alongside the asymptotic approximation based on the contribution of the caterpillar tree, taking the $\log _2 \log$ of these quantities to produce a comparable scale to Figure 2. In the figure, we observe that even for quite small *n*, the exact mean and variance are closely approximated by the asymptotic $\pi _n c_{n-1}$. The mean and variance are greatest for the uniformly random leaf-labeled trees, for which $\pi _n \sim \sqrt{\pi } ( n^{3/2} )(0.5^n)$ (12), followed by the uniformly random unlabeled unordered trees, with asymptotic approximation $\pi _n \sim \kappa n^{3/2} \rho ^{n} \approx 3.13699 (n^{3/2})(0.40270^n)$ (13). For the Yule–Harding model, caterpillars are least probable (14).

## Discussion

We have analyzed the Colijn–Plazzotta rank of rooted binary trees, showing that the rank of a tree is largely determined by its height. Indeed, the ranking proceeds through all trees of a given height *h* before moving on to trees of height $h+1$. We have also obtained asymptotic properties of the trees under three different models for selecting random trees, finding in particular the asymptotics of ${\mathbb {E}}\{\log _2 \log f(\tau _n) \}$ for random trees $\tau _n$. The asymptotic mean and variance of the CP rank across trees with *n* leaves depend only on the probability and CP rank of the *n*-leaf caterpillar, as the product of the probability and the CP rank of the caterpillar grows faster than the next-highest rank. A summary of mathematical results appears in Table 3.

Numerical investigations clarify a pattern observable in the mathematical results, namely that the “uniform” model—uniformly random leaf-labeled trees—has CP ranks greater than the Yule–Harding model on leaf-labeled trees (Figures 2–4). This observation can be viewed as a consequence of the greater probability of the caterpillar shape in the uniform (12) than in the Yule–Harding model (14).

It has been suggested that CP rank can serve as a measure of tree balance and imbalance in empirical studies (Fischer et al. 2023; Rosenberg 2021). We have found that as *n* grows, the CP rank of the caterpillar grows so fast that for both the uniform and Yule–Harding models on leaf-labeled trees, the mean CP rank across trees with *n* leaves is asymptotically determined by the contribution of the caterpillar. Hence, as a balance statistic beyond the smallest tree sizes, the use of CP rank $f(\tau )$ would amount primarily to distinguishing caterpillars from non-caterpillars. A potentially more suitable statistic is $\log _2 \log f(\tau )$, which places the CP ranks of different trees on a similar scale. Due to its extremely large values, the CP rank has been omitted from an empirical comparison of tree balance statistics (Kersting et al. 2025); we suggest that this problem could be resolved by including its double-logarithm in its place.

The results have been obtained by connecting studies of CP rank as a quantity of mathematical phylogenetics to the extensive literature on tree height in studies grounded in theoretical computer science. As has been demonstrated here, such applications of theoretical computer science results on tree properties have the potential to provide solutions to unsolved problems in mathematical phylogenetics.

Although we have obtained the asymptotics of the mean and variance of the CP rank under the uniform and Yule–Harding models—the two models for which the mean and variance were noted by Fischer et al. (2023) as open problems—we have not commented on the *exact* mean and variance. For practical applications of CP rank, an understanding of the asymptotics likely suffices, but we note that the precise determination of the mean and variance of the CP rank remains an open problem.

## Data Availability

The study has no associated data.
